# Assessing the contribution of active somatosensory stimulation to self-acceleration perception in dynamic driving simulators

**DOI:** 10.1371/journal.pone.0259015

**Published:** 2021-11-18

**Authors:** Mattia Bruschetta, Ksander N. de Winkel, Enrico Mion, Paolo Pretto, Alessandro Beghi, Heinrich H. Bülthoff

**Affiliations:** 1 Department of Information Engineering, University of Padova, Padova, Italy; 2 TU Delft, Cognitive Robotics Delft, Delft, Netherlands; 3 Department of Perception, Cognition, and Action, Max Planck Institute for Biological Cybernetics, Tübingen, Germany; 4 Virtual Vehicle Research, Graz, Austria; Anglia Ruskin University, UNITED KINGDOM

## Abstract

In dynamic driving simulators, the experience of operating a vehicle is reproduced by combining visual stimuli generated by graphical rendering with inertial stimuli generated by platform motion. Due to inherent limitations of the platform workspace, inertial stimulation is subject to shortcomings in the form of missing cues, false cues, and/or scaling errors, which negatively affect simulation fidelity. In the present study, we aim at quantifying the relative contribution of an active somatosensory stimulation to the perceived intensity of self-motion, relative to other sensory systems. Participants judged the intensity of longitudinal and lateral driving maneuvers in a dynamic driving simulator in passive driving conditions, with and without additional active somatosensory stimulation, as provided by an Active Seat (AS) and Active Belts (AB) integrated system (ASB). The results show that ASB enhances the perceived intensity of sustained decelerations, and increases the precision of acceleration perception overall. Our findings are consistent with models of perception, and indicate that active somatosensory stimulation can indeed be used to improve simulation fidelity.

## 1 Introduction

Dynamic driving simulators are nowadays a widely used tool within automotive companies, both in R&D and in production departments. Originally designed for race applications, their role is becoming crucial also for autonomous driving and Advanced Driver Assistance Systems (ADAS), where human-machine interactions (HMI) can be analyzed in a way that ensures safety and repeatability.

In dynamic driving simulators, the driving experience is reproduced by combining visual information, generated by graphical rendering, with inertial stimuli, generated by translations and rotations of the motion platform. The strategies that have the aim of translating vehicle motion into feasible simulator motions are called Motion Cueing Algorithms (MCA). Since limitations of the simulator’s motion envelope preclude a 1:1 reproduction of the desired accelerations in most cases, MCA typically reproduce low-frequency dynamics by down-scaling, tilt-coordination, and a continuous calling back to the initial position (washout); thereby reducing the actual motion of the simulator, while trying not to compromise the subjective realism of the motion cues [1]. Although efforts are made to improve upon this washout strategy (see e.g., work on Model Predictive Control [2–6]), the inherent limitations of a motion simulator’s envelope cannot entirely prevent missing cues, false cues, and/or scaling errors. These limitations may in turn negatively affect the perceived realism and immersion in the simulation, and may even trigger motion sickness [1, 7–9].

In real-life, a vehicle’s inertial motion results in stimulation of different sensory systems of the driver throughout the body; notably the vestibular and somatosensory senses. The vestibular system comprises the semicircular canals and otoliths of the inner ear, which are directly sensitive to angular and linear accelerations, respectively. The somatosensory system consists of various sensory neurons, distributed throughout the body, that are indirectly responsive to physical motion. These neurons respond to force cues that are generated when, for instance, the body is pressed into the seat or safety belts during accelerations, due to its inertia. Consequently, it is possible to separately recreate somatosensory stimulation by actively providing specific pressure cues. This idea has been explored in flight simulators, in the form of the g-seat [10–13] and in driving simulators, in the form of the Active Seat (AS) and Active Belts (AB) [14], and has the potential to reproduce part of the low-frequency sustained accelerations that generally lack in compact motion simulators with a limited workspace [15].

There is a considerable neuroscientific literature that addresses how the human brain constructs a unified conscious experience out of multisensory cues. Several studies have shown that observers create a robust percept about the environment by weighing multiple sources of sensory information based on their reliability [16, 17]. Even though statistically optimal integration does not always apply [18], there is evidence that weighing is also involved in the perception of self-motion: for instance, visual and inertial cues to heading [19–23] and orientation [24–26] appear to be integrated optimally, provided that discrepancies between the signals remain within a certain range [27–29]. Similar findings have been reported for the perceived magnitude of angular [30] and linear motion [31–33]. Providing a detailed characterization of how multisensory stimuli interact in self-acceleration perception during the driving task is also a topic of current interest [34]. However, whereas visual-inertial interactions have thus received considerable attention in both real and simulated driving [35, 36], the relative contribution of somatosensory cues to the overall motion percept is, to the best of our knowledge, poorly understood. In particular, the skin receptor dynamics, that are of interest in the application at hand, are difficult to measure and quantify [34]. In the present study, we investigated how the perception of linear and angular motions is affected by active somatosensory stimulation. Our experimental setup consisted of a hexapod motion simulator with a head-mounted display (HMD) and an Active Seat (AS) and Active Belts (AB) integrated system (ASB). The ASB system consists of a seat equipped with a pneumatic system that can be used to recreate pressure cues acting on the body during accelerations by emulating the contact between seat, belts and body that result from inertial accelerations. We compared the perceived motion resulting from visual and platform movements (*visual-vestibular motion* condition) to perception resulting from visual and platform movements with additional somatosensory cues (*somatosensory augmented motion* condition).

We determined the effects of the active somatosensory stimulation in two different driving maneuvers: a straight line and a curve, that are characterized by longitudinal and lateral accelerations, respectively. Generally speaking, longitudinal accelerations are generated by forward and backward surges of the platform, whereas lateral accelerations are provided through left and right sways. Because longitudinal and lateral motions stimulate different groups of somatosensory neurons, we evaluated the ASB contribution in two experiments, respectively. In both experiments, participants were passively transported in an autonomous (virtual) vehicle. They were only asked to perform a Magnitude Estimation (ME) task, to provide a numerical estimate of the acceleration intensity, and a two-Interval Forced Choice (2IFC) task, to compare the acceleration intensities.

## 2 Materials and methods

### 2.1 Ethics statement

The experiments were carried out in accordance with the declaration of Helsinki. All participants provided written informed consent prior to participation. The experimental protocol was approved by the ethical committee of the medical faculty of the Eberhard-Karls University in Tübingen, Germany, reference number 800/2018BO1.

### 2.2 Participants

In total, ten different volunteers took part in the experiments (3 females, mean age 29.2 years, standard deviation 4.1). In accordance with motion platform safety guidelines, participation was limited to people measuring at most 1.95m long and weighing under 100 kg. All participants were familiar with the simulator and reported not to be susceptible to motion sickness and claustrophobia. Participants were assigned to one of two experimental groups: the first group experienced only cornering maneuvers (6 participants, 1 female, mean age 28.8 years, sd 4.2), whereas the second group experienced only car braking maneuvers (4 participants, 1 female, mean age 29.8 years, sd 4.5). Details of the experiments are given below, in the section Task and Stimuli. One participant participated in both experiments (i.e., both the longitudinal and lateral cases). One other participant (P05, longitudinal condition) was excluded from the formal analysis. During the debriefing, this participant reported to have been confused by the presence of active somatosensory stimulation and stated to have used different cognitive strategies to solve the tasks in the two conditions. This resulted in worse performance with active somatosensory stimulation.

### 2.3 Apparatus

Physical acceleration cues were generated using an eMotion-1500 hexapod-based motion system (Bosch Rexroth, 2015) at the Max Planck Institute for Biological Cybernetics. This platform is specifically designed for human-occupied vehicle simulations (aircraft, cars, trucks, buses, military vehicles, railway vehicles, etc.), entertainment, or research and development. The dynamic motion simulator was controlled using Simulink and MATLAB software (The MathWorks Inc., Natick, Massachusetts, United States). As the experiment is based on a passive driving experience, the platform was moved using motion references computed off-line. The proposed maneuvers are composed by a single event with a limited duration (below 10 seconds), hence the specific methodology used for the Motion Cueing does not play a critical role in the experiment design. However, to guarantee the possibility to recreate the experiment, the commercial software VI-DriveSim that includes VI-MotionCueing (VI-grade GmbH [37]) was used to generate the motion profiles. Tuning of the motion cueing was done following the guidelines recommended in the manual and refined during the study’s pilot phase to reach a subjectively optimal experience and was equal for all participants. A list of the parameters that have been modified w.r.t. the default tuning is reported in Tables 4 and 5 in S1 Appendix.

**Fig 1:**
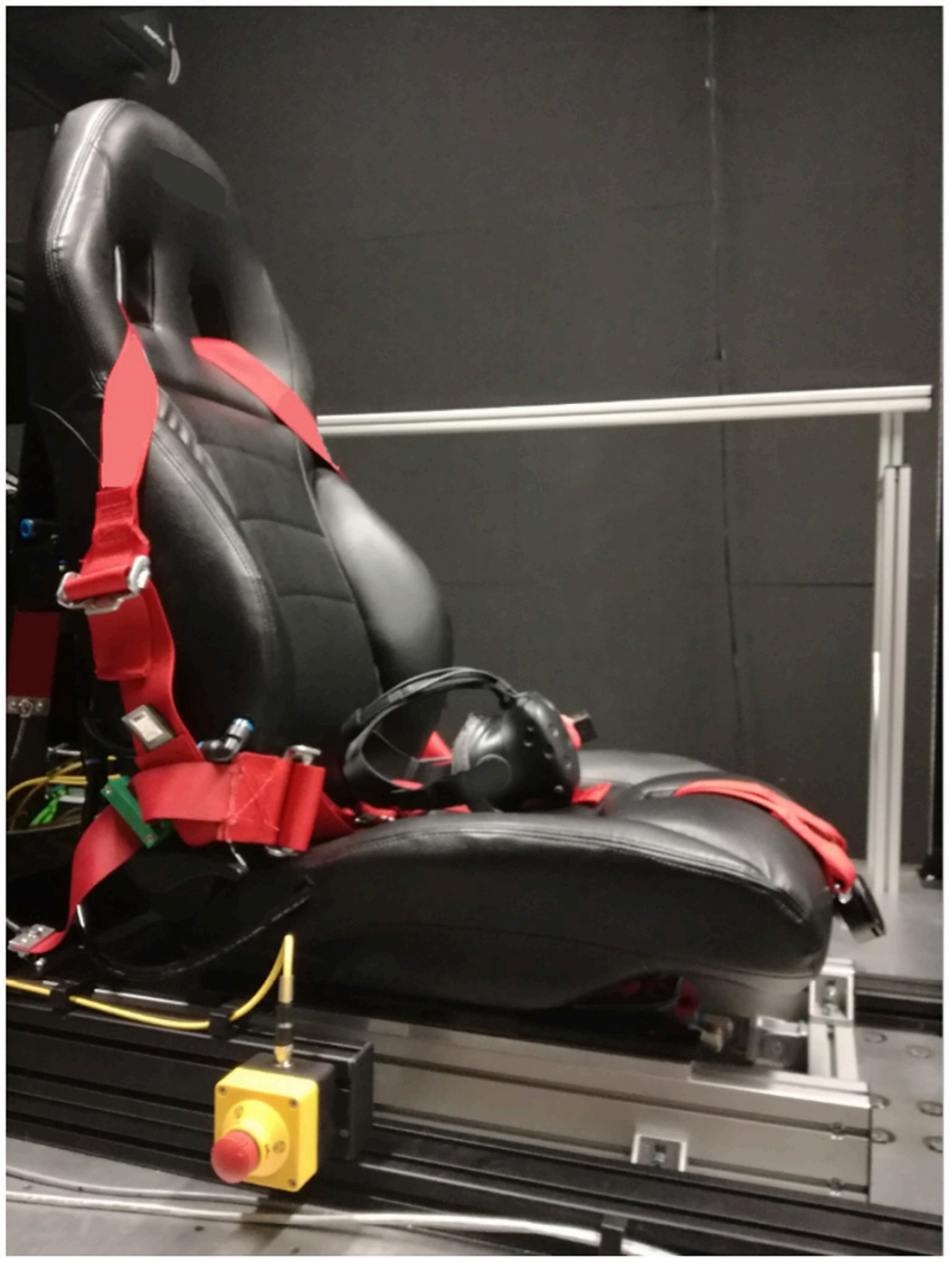
Active seat. The AS mounted on the motion platform with 6-point AB and Head-Mounted Display (HMD).

**Fig 2:**
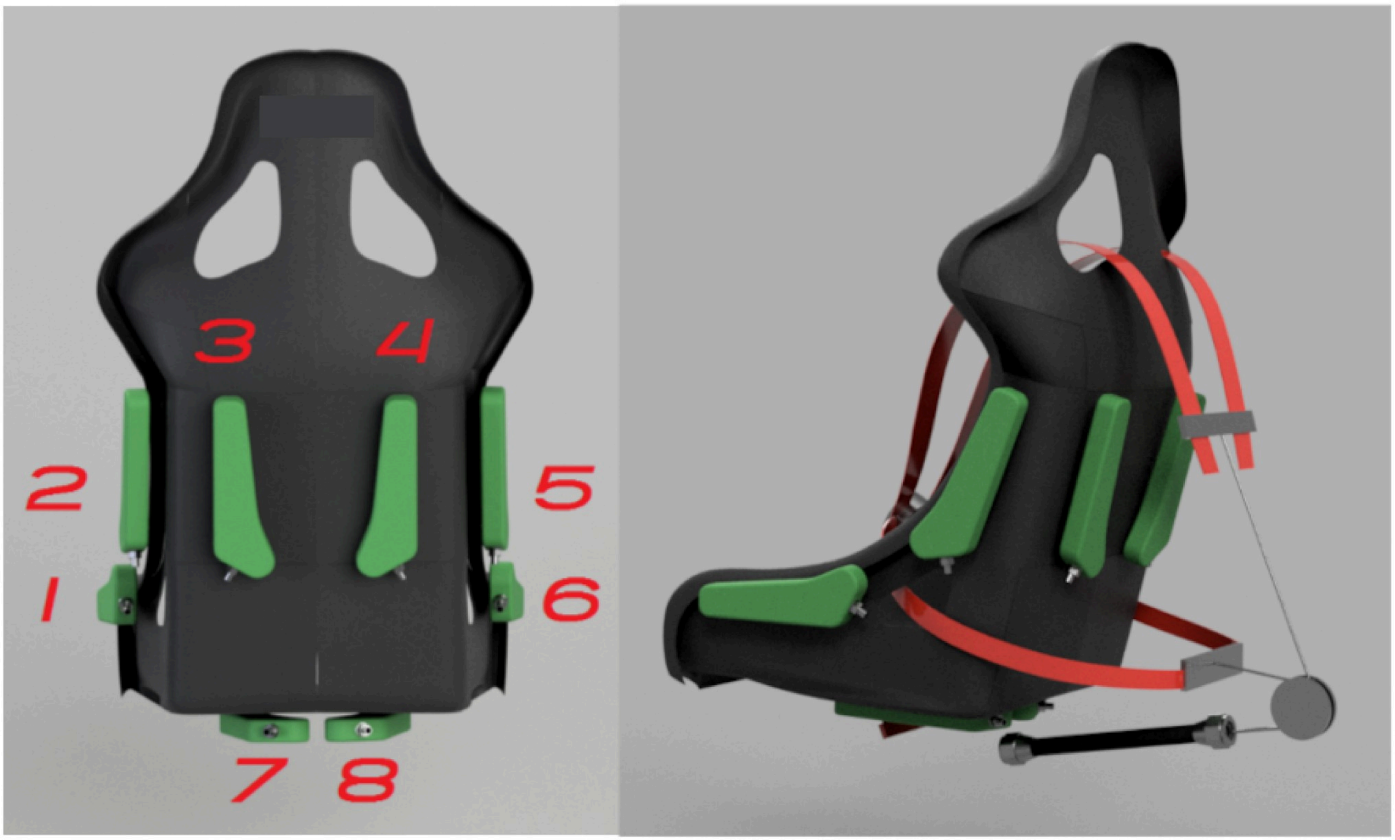
ASB system: The left picture shows the position of the bladders, whereas the picture on the right shows the belts tensioned by a muscle.

An Active Seat (AS) and 6-points Active Belts (AB) were used in combination (ASB) to provide active somatosensory stimulation (VI-grade GmbH). The seat was rigidly mounted on the motion platform and is shown in Fig 1. The AS part consists of eight air bladders placed in specific locations (see Fig 2, left panel). The bladders can be inflated in proportion to vehicle accelerations. Bladders 2 and 5 provide pressure to the lower sides of the trunk of the body (roughly at the location of the external oblique muscles); bladders 1 and 6 to the sides of the upper legs during lateral accelerations (vastus lateralis muscles); bladders 3 and 4 to the lower back during positive longitudinal acceleration (latissimus dorsi muscles) and, finally, bladders 7 and 8 provide pressure to the buttocks and rear of the upper legs (gluteus muscles) for accelerations in the vertical direction. The bladders are designed to have a distributed contact area and are placed such that they can be used to mimic pressure cues generated in a real car. Proportional valves are used to allow for a progressive and continuous variation of pressure, which can be varied in the range 0–2 bar.

The AB are commanded by a pneumatic muscle (see Fig 2, right panel), and are intended to provide the driver with pressure cues to the torso (roughly the pectoralis major and trapezius muscles) during longitudinal acceleration of the vehicle.

The ASB system is controlled by VI-DriveSim 18.1 software. This software allows the tuning of the parameters directly from a user interface. As for the motion cueing, the editable parameters have been tuned in the study’s pilot phase to provide a subjectively optimal experience and were equal for all participants. The values used are given in tables in S1 Appendix.

**Fig 3:**
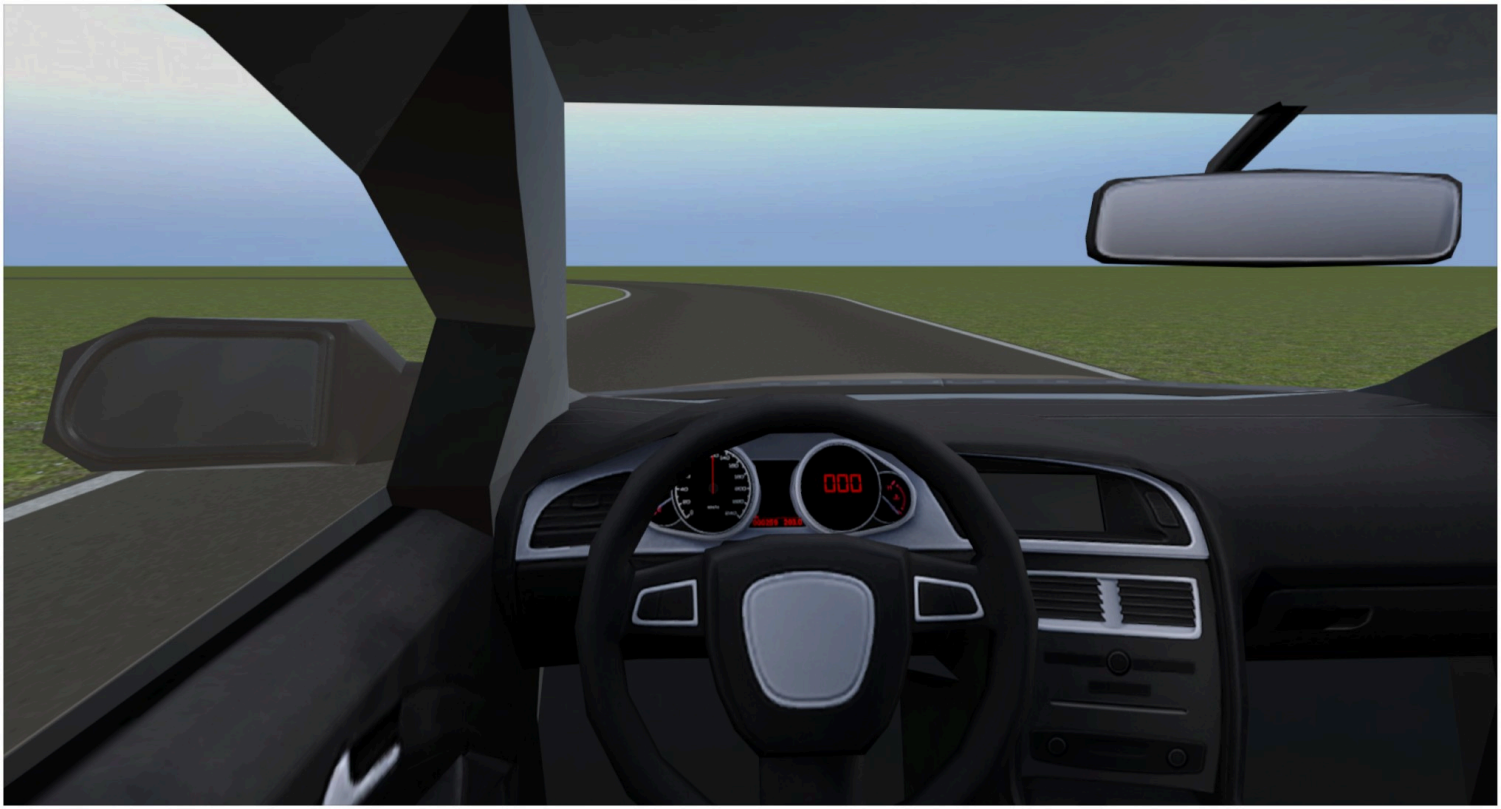
View from HMD. Screenshot of the view participants had from the inside of the virtual car.

Visual stimuli were presented using a Vive head-mounted display (HMD) (HTC, New Taipei City, Taiwan). The screens each have 1080 × 1200 pixels and a refresh rate of 90fps. The HMD was used to present participants with visual motion stimuli that depicted a view from the inside of a car driving over a straight road or a leftward curve, in the longitudinal and lateral case, respectively, as shown in Fig 3. To reduce the possibility that a participant focused on reference points to perform the task there were no elements in the scene such as trees or buildings. The scenes were generated using the Unity game engine (Unity Technologies, San Francisco, United States). Head motions were tracked, corrected for platform motion, and reproduced in the virtual environment using the Vive ‘lighthouse’ tracking boxes with OpenVR-InputEmulator [38].

Sounds generated by the motion platform were masked using headphones (Plantronics, California, United States) that actively cancelled outside noise and played back simulated engine sounds. In addition, participants wore earplugs with a 33dB signal-to-noise ratio (Honeywell Safety Products, Roissy, France).

Verbal responses (ME task) were noted by the experimenter; binary responses (2IFC task) were collected using an Xbox controller (Microsoft, Redmond, WA, United States)

### 2.4 Task and stimuli

The study was designed to determine the contribution of somatosensory cues, as delivered by the ASB system, to the perception of accelerations in a dynamic driving simulator. Because the contribution of the AS and AB subsystems and the stimulated areas of the body differ for lateral and longitudinal accelerations, we created two experiments in which cornering (lateral motion) or braking maneuvers (longitudinal motion) were presented, respectively. Participants were randomly assigned to either experiment.

Similar to [39], who used two separate tasks to determine the relative contributions of accelerations and jerk to the perceived intensity of motions, we used (1) a Magnitude Estimation (ME) task to assess *accuracy* of perception, and (2) a 2-Interval Forced Choice (2IFC) task to assess *precision* of perception. Both tasks were used in each part of the experiment.

In the ME task, participants were presented with driving maneuvers with different acceleration intensities and were asked to provide verbal estimates of their intensity; in the 2IFC task, participants were presented with two successive motions on each experimental trial, and asked to judge which of the two stimuli was more intense.

We then evaluated the effects of active somatosensory stimulation by comparing perceived motion in visual-vestibular motion conditions, where combinations of visual stimulation and platform movements (*visual-vestibular motion*) were presented, to perceived motion in augmented conditions, with active somatosensory cues (*somatosensory augmented motion*) in addition to the visual and platform movements.

#### 2.4.1 Tasks

*2.4.1.1 Magnitude estimation*. In the ME tasks, participants were required to provide verbal estimates of different acceleration intensities of the proposed driving maneuvers. For both cornering and braking maneuvers, each of the seven stimulus intensities was presented three times. As a reference for their responses, participants received three consecutive training trials at the beginning of the experiments. These were chosen to have exactly the medium intensity within the two ranges: the peak acceleration intensity of the training stimuli for the braking maneuvers was 1.75 m/s^2^, and the peak acceleration intensity of the training stimuli for the cornering maneuvers was 5 m/s^2^. Participants were instructed to attribute the arbitrary number ‘100’ to the intensity of the training trials. In total, participants performed 24 ME trials for the *visual-vestibular motion* condition, and 24 for the *somatosensory augmented motion* condition. The order of stimuli was randomized.

*2.4.1.2 2-interval forced choice*. Although the ME method is thought to yield insights in the relationship between stimulus intensity and perceived intensity, it does not provide an unambiguous scale because idiosyncratic internal representations on the unit of acceleration may differ, and it does not provide accurate information on the precision of perception because numerical estimates are subject to cognitive biases such as a rounding off to the nearest decade [40]. Therefore, participants were asked to also perform a 2IFC task.

In a 2IFC task, participants experience two successive motions for each experimental trial, designated ‘reference’ and ‘comparison’. The participants’ task is to judge which of the two stimuli was more intense. The reference stimulus has a peak intensity that is kept constant among trials, whereas the peak intensity of the comparison stimulus is chosen from the range of values around the reference. By obtaining repeated judgments for several comparison intensities, it is possible to determine how the proportion of responses (or their probability) where the comparison is judged more intense than the reference depends on the intensity of the comparison stimulus, which in turn can be used to estimate perceptual precision. Each reference-comparison pair was presented 10 times, resulting in eighty trials for the M and S conditions, making for a total of 160 2IFC trials per participant. This number was the same for the cornering experiment and for the braking experiment. The order of trials and the order of reference and comparison motion within trials were randomized.

By combining the ME and 2IFC tasks, we aimed to obtain a fair characterization of perceived motion.

#### 2.4.2 Stimuli

The acceleration ranges are based on [41] and were tailored to achieve a realistic driving experience during pilot experiments. The specific values were selected to cover a range of accelerations that is commonly experienced during normal driving, and that provides a vestibular-somatosensory stimulation above the absolute perception threshold when reproduced on the simulator. In terms of subsystem capacity, the average ratio of AS÷AB at the peak intensity of the lateral maneuvers was 6%÷60%; this ratio was 48%÷1% during longitudinal maneuvers.

*2.4.2.1 Cornering maneuvers*. Trajectories were generated by imposing the same duration of the stimulus (5s) and the same curve angle (90 degrees), while varying the acceleration intensity, thereby manipulating the curvature radius and the travel velocity.

**Fig 4:**
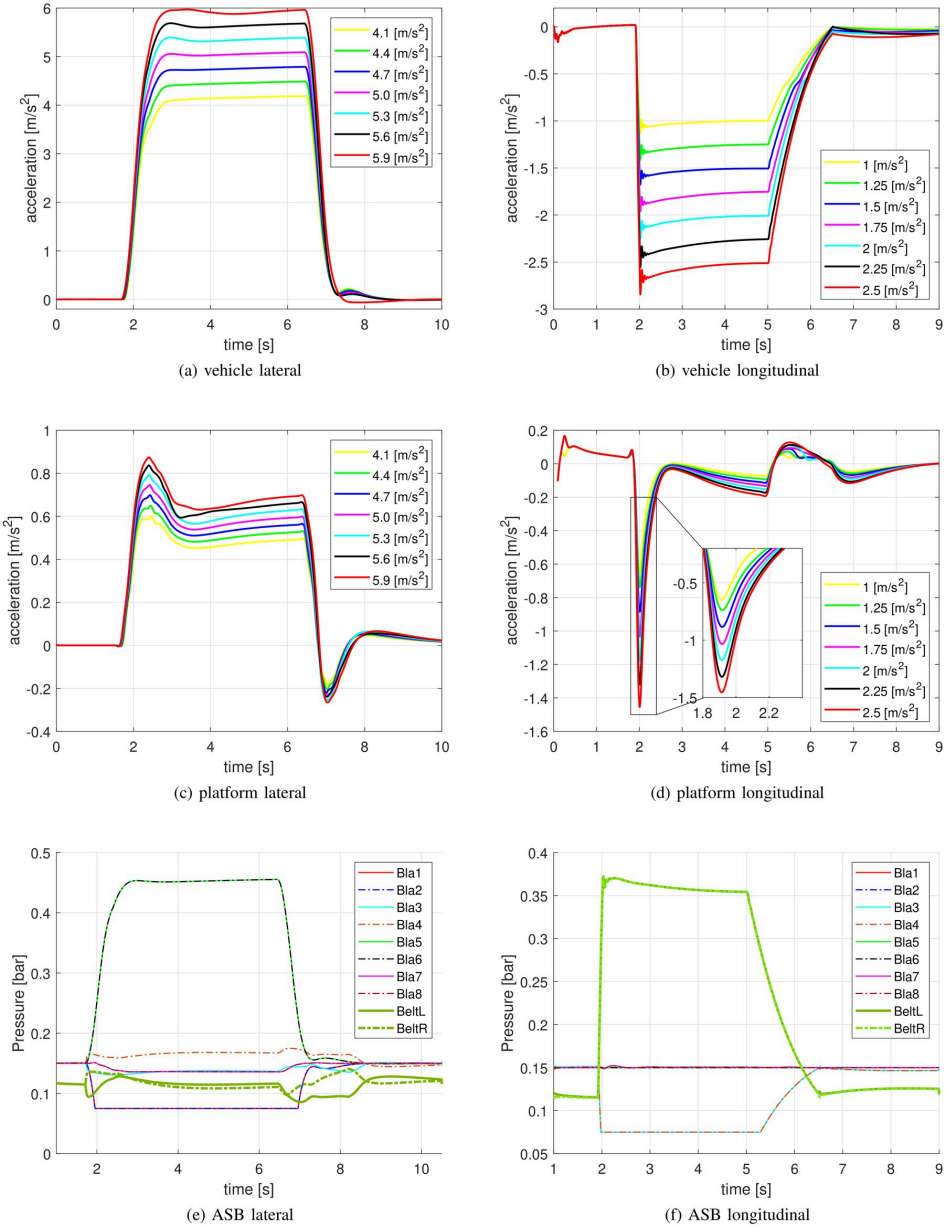
Desired motion and stimulation profiles. Top row: vehicle acceleration profiles for the lateral (a) and longitudinal (b) case; middle row: platform accelerations including tilt coordination for lateral (c) and longitudinal (d) case; bottom row: pressure cues generated by the ASB, in the 5.0m/s^2^ lateral (e) and 1.75m/s^2^ longitudinal (f) condition. Legends report the values of the acceleration peaks of the vehicle during the maneuver for the top and middle row. In the bottom row, the legend specifies the ASB actuator, as in Fig 2.

For the ME task, accelerations were varied between 4.1 and 5.9 m/s^2^ in seven equidistant steps (Fig 4(a)). The corresponding platform accelerations, which include tilt coordination contribution, obtained from the VI-DriveSim software, are shown in Fig 4(c); exact quantification of the pressure cues generated by the ASB system is shown in Fig 4(e) for the 5.0m/s^2^ maneuver.

For the 2IFC task, the reference intensity was 5.2 m/s^2^, and the comparison intensities varied between 4.5 − 5.9 m/s^2^ in nine equidistant steps, but omitting 5.2 m/s^2^ as a comparison value, resulting in eight comparison stimuli. The profiles for vehicle accelerations, platform accelerations and displacements were identical to those of the ME-task except for their scaling, and are therefore not shown separately.

It may be noted that the cueing software introduced minor false cues (e.g., the negative accelerations around 6–8 s at the end of the profiles in Fig 4c). However, as these deflections are small and because participants did not report noticing them, nor any dizziness/sickness, we believe they did not have any notable effects on our findings.

*2.4.2.2 Braking maneuvers*. The trajectories featured a sustained deceleration (3s).

For the ME task, the range of peak accelerations was varied between 1 and 2.5 m/s^2^, in seven equidistant steps (Fig 4(b)). The corresponding platform accelerations, which include tilt coordination contribution, are shown in Fig 4(d) and 4(f) shows an exact quantification of pressure exerted to the body by the ASB system for the maneuver with a 1.75m/s^2^ acceleration peak.

For the 2IFC task reference intensity was 2 m/s^2^, and the comparison magnitudes ranged between 1.5 − 2.5 m/s^2^ in nine equidistant steps, but omitting 2 m/s^2^ as a comparison value, resulting in eight comparison stimuli. As for the cornering experiment, the profiles for vehicle accelerations, platform accelerations and displacements were identical to those of the ME-task except for their scaling, and are therefore not shown separately.

The lateral and longitudinal maneuvers are designed to produce similar acceleration peaks on the platform while exploiting all the available working space.

### 2.5 Procedure

After participants had understood the instructions and safety requirements and had given their written informed consent, the experimenter guided them to the simulator platform and seated them in the AS. Then, the AB were put in place, as were the ear plugs, the headphones, and the HMD. The experimenter asserted that the belts were properly tightened and that the thighs of the participants rested on the seat. When participants were properly installed, they were given the controller, which they used to provide responses and to advance trials at their own pace.

Before the experiment started, participants were given three training trials to familiarize with the overall procedure and apparatus. At the end of the task, participants were debriefed and asked to fill out a questionnaire with questions regarding their subjective experience of the ASB contribution.

Altogether, the experiments took approximately three hours to complete per participant, including instructions, training sessions and pauses. Each participant completed the experiments in two sessions, both combining ME and 2IFC, one with the whole setup active and the other with ASB switched off. The sessions lasted one and a half hour each and were performed on two different days.

Assignment of participants to the cornering or braking maneuver; the order of the visual-vestibular motion and somatosensory augmented motion; and the order of ME and 2IFC tasks were randomized.

### 2.6 Data analysis

The data analyses were designed to determine how the addition of active somatosensory stimulation affected perception, in comparison to a visual-vestibular motion condition with visual and platform motion only. Because the ME and 2IFC tasks yield different types of response data (continuous and dichotomous, respectively), we performed separate analyses for the two measures. Data acquired are collected in SI 1 and 2.

For ME data, a linear mixed-effect model was fitted to the data as a psychometric function. In this model, we describe responses on the ME task *y*_*ME*_ in terms of fixed effects that were common to all participants and random effects that account for additional individual variations in these effects. The model has terms for the categorical dummy variables take on values of 0 or 1 to indicate the absence or presence of some categorical effect. variables ‘maneuver’ *man* and ‘condition’ *con*; a term for the continuous variable ‘peak intensity’ *int*, and terms for the interactions between these variables, which allow accounting for non-additive effects of different variables. In addition, we allow for variation between participants *par* by including random effects with the same structure as the fixed effects model.

In Wilkinson notation [42], the model fitted to the data of the ME task can then be defined as:

$$\begin{array}{c} y_{ME}∼1+man\times con\times int+\left(1+man\times con\times int|par\right). \end{array}$$ (1)

In the 2IFC case, responses are binary, which means a linear regression model cannot be used. Instead, a *probit* mixed-effect model has been fitted to the data. *Probit* is a generalized regression model where the response variable can have two possible outcomes only. In Wilkinson notation [42], the model was defined as:

$$\begin{array}{c} y_{2IFC}∼\Phi \left(-1+int+int:\left(man\times con\right)+\left(-1+int+int:\left(man\times con\right)|par\right)\right). \end{array}$$ (2)

Here, Φ is the Cumulative Distribution Function (CDF) of the standard normal distribution. Note that this model includes effects for ‘peak intensity’ and all its interactions with ‘maneuver’ and ‘condition’, but lacks an intercept (‘-1’) and main effects for ‘maneuver’ and ‘condition’ (i.e, the possibility for the intercept to vary between conditions). This is so because, in a 2IFC task, the intercept is fixed to the value of the reference stimulus, which did not vary between conditions. Consequently, for the 2IFC task, we only account for variations in the slope of the psychometric function as a result of experimental manipulations.

Models were fitted to the data using Maximum-Likelihood Estimation. We assessed the (adjusted) coefficient of determination ($R_{adj}^{2},R^{2}$, respectively) as an indicator of the quality of our fitted models. These statistics indicate the proportion of variance in the data explained by the model. P-values were computed for each regressor term in order to determine whether it provided a statistically significant effect: we accept a 5% chance of rejecting a true null hypothesis, that is, we accept there is an effect of a particular variable that is actually due to chance.

Because we are interested only in the general effect of the presence of active somatosensory stimulation, we do not explicitly consider the random effects in our analyses.

## 3 Results

### 3.1 Magnitude estimation

**Fig 5:**
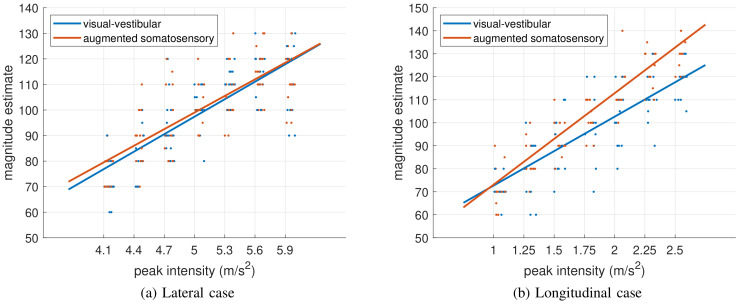
Data acquired in the ME task for the cornering (left panel, (a)) and brake maneuver (right panel, (b)) cases. Light blue and orange dots represent individual responses collected in the M and S conditions, respectively. A small amount of random noise was added to the x-coordinate of individual data points to improve their visibility. Lines are the four fitted psychometric functions obtained by model (1), which describe the four cases in which participants have been split, i.e. the braking and the cornering maneuver, both experienced with or without ASB active. The results suggest that the bladders of the ASB system do not increase perceived intensity in curves, but the belts do increase perceived intensity in braking maneuvers. No unit is provided for the y-axis as magnitude estimates are expressed on a subjective scale.

Verbal estimates collected in the ME tasks and the fitted psychometric functions are shown in Fig 5(a) and 5(b), for the lateral and longitudinal case, respectively. Overall, the model provided a fair approximation of the data, as evidenced by *R*^2^ = 0.72 and $R_{adj}^{2}=0.71$.

The estimated coefficients for the psychometric functions and the associated p-values are presented in Table 1.

**Table 1:** Fixed-effects coefficients and associated p-values for the ME-task psychometric function.

Regressor variable	Coefficient	pValue
‘(Intercept)’	$\beta_{0_{ME}}=42.751$	1.1458e-21
*man*	$\beta_{m_{ME}}=-59.354$	9.0245e-10
*con*	$\beta_{c_{ME}}=-9.3802$	0.13202
*int*	$\beta_{i_{ME}}=29.95$	2.4799e-36
*man* × *con*	$\beta_{mc_{ME}}=16.735$	0.18513
*man* × *int*	$\beta_{mi_{ME}}=-7.1543$	0.0094258
*con* × *int*	$\beta_{ci_{ME}}=9.7987$	0.016781
*man* × *con* × *int*	$\beta_{mci_{ME}}=-10.945$	0.016936

As the p-value for the *int* coefficient is smaller than 0.05, we can consider the effect significant. This means that responses on the ME task depend on the peak intensity of the stimulus, thereby confirming that participants were able to perform the task.

Significant p-values in *man* × *int*, *con* × *int*, *man* × *con* × *int* indicate that there are interaction effects: as can be seen in Fig 5(a) and 5(b), the slope of the psychometric function differs between experimental conditions. The significance of the effect of *man* combines with significant effect for *man* × *int*, which together reflect the fact that people were asked to associate the arbitrary value of 100 to the middle value of the range of intensities presented, whereas the range of intensities differed between maneuvers.

Notably, the slope of the psychometric function is steeper in the S condition than in the M condition, but only for longitudinal motion stimuli.

### 3.2 Two-interval forced choice

**Fig 6:**
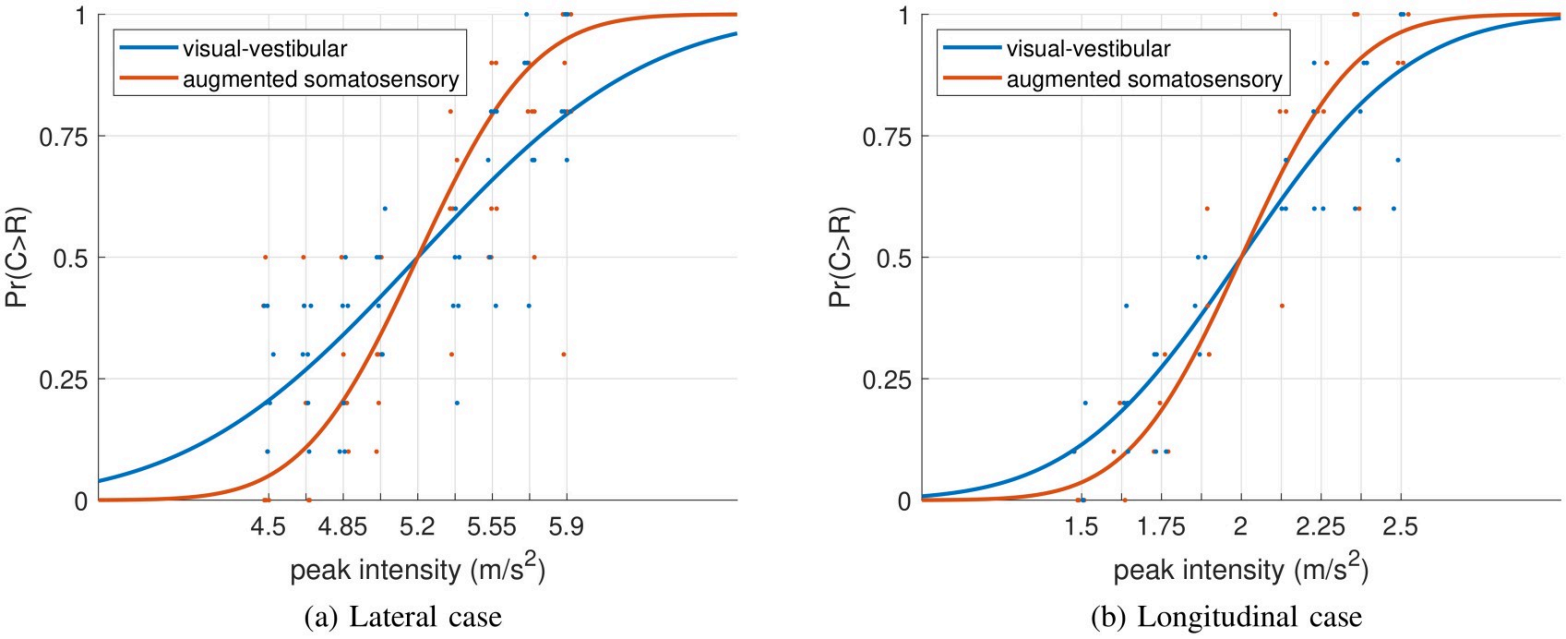
Data acquired in the 2IFC task for the lateral (left panel, (a)) and longitudinal (right panel (b)) cases. Light blue and orange dots represent the proportion of trials where the comparison stimulus was judged larger than the reference by an individual participant for each comparison stimulus intensity, for responses collected in the M and S conditions, respectively. A small amount of random noise was added to the x-coordinate of individual data points to improve their visibility. Lines are the four fitted psychometric functions obtained through model (2). The results suggest that active somatosensory stimulation increases discrimination sensitivity.

Binary responses and fitted psychometric functions task are shown in Fig 6(a) and 6(b). Overall, the model provided a fair explanation of the data, as evidenced by *R*^2^ = 0.53 and $R_{adj}^{2}=0.53$.

Table 2 shows the estimated fixed-effects coefficients and the associated p-values.

**Table 2:** Fixed-effects coefficients and associated p-values for the 2IFC-task psychometric function.

Regressor Variable	Coefficient	pValue
*int*	$\beta_{i_{2IFC}}=2.4072$	3.1376e-09
*man* × *int*	$\beta_{mi_{2IFC}}=-1.2337$	0.011969
*con* × *int*	$\beta_{ci_{2IFC}}=1.1751$	0.00343
*man* × *con* × *int*	$\beta_{mci_{2IFC}}=-0.85309$	0.10061

As the p-value related to *int* is lower than 0.05, we can conclude that the probability of response that the comparison stimulus was larger than the reference (*C* > *R*) increases with stimulus peak intensity, which means that participants were able to perform the task.

The interaction effects between maneuver and peak intensity (*man* × *int*) and between condition and peak intensity (*con* × *int*) were also significant. These findings indicate that the slope of the psychometric function differs depending on whether people performed the lateral or longitudinal task, and depending on whether there was active somatosensory stimulation, respectively.

Notably, the slope of the psychometric function was steeper when there was active somatosensory stimulation, and this effect was consistent between maneuvers (i.e. the effect for *man* × *con* × *int* was not significant).

### 3.3 Debriefing phase

After completing the main tasks, participants were asked to answer the following questions regarding their subjective experiences:

1. “Do you think the presence of the ASB system improves the realism of the simulation?” (0 no, 1 yes);
2. “Did you feel immersed; as if you were in a real car?” (0 no, 1 without ASB, 2 with ASB);
3. “Did the ASB system help you in distinguishing the different stimulus intensities?” (0 no, 1 yes);
4. “What do you think is the source of information that helped you most in performing the task?” (0 visual motion, 1 platform motion, 2 the ASB system, 3 other, namely …);
5. “Did you experience the ASB-system as a natural addition to the motion simulation?” (0 no, 1 yes).

The responses are shown in Table 3, and can be summarized as follows: (1) all participants considered the addition of the ASB to improve the realism of the simulation. (2) Nine participants (9/10) indicated that they felt more immersed in the simulation when ASB was present. (3) Eight (8/10) participants thought that ASB helped them to distinguish motion intensities. Participants considered platform motion and ASB as the main sources of information exploited to perform the tasks, whereas only three participants declared that they used also visual information. All participants except P05 (whose data were excluded from the quantitative analyses, see 2.2) considered the ASB-system a natural addition to the motion simulation. Given the high rating of realism provided by participants, it is unlikely that any interference or incompatibility has arisen between platform motion and somatosensory cues. During the debriefing, some participants suggested that there was room for improvement regarding the naturalness of the stimuli.

**Table 3:** Participants’ answers on the questionnaire. The values for ‘case’ refer to whether participants were in the experiment with longitudinal (0) or lateral (1) motions.

Participant	Case	Q1	Q2	Q3	Q4	Q5	Participant	Case	Q1	Q2	Q3	Q4	Q5
P01	0,1	1	1	1	1,2	1	P06	1	1	0	1	0,1,3	1
P02	1	1	1	1	0,2	1	P07	1	1	1	1	1,2	1
P03	1	1	1	0	0,1	1	P08	0	1	1	1	1,2	1
P04	0	1	1	1	2	1	P09	0	1	1	1	2	1
P05	0	1	1	1	1,2	0	P10	1	1	1	0	1	1

## 4 Discussion

The present work was designed to gather a first quantification of the contribution of active somatosensory stimulation to dynamic driving simulations. We assessed this by asking participants to rate the intensity of their motion percepts and to perform a discrimination task for visual-vestibular motion driving simulation scenarios (platform and visual motion) and for an augmented condition that featured additional active somatosensory stimulation. Because of the contribution of the AS and AB subsystems and the parts of the body that are stimulated differ for lateral and longitudinal motions, we performed separate experiments for both cases.

In our analysis, the data from the longitudinal (four participants) and lateral (six participants) maneuvers was pooled. Hence, assessments of ASB effects common to the two maneuvers were based on observations in ten participants [51–55].

Intensity ratings were obtained using a Magnitude Estimation (ME) paradigm. In this paradigm, participants attribute scores to stimuli of different intensities, with reference to a standard that was presented at the outset of the experiment. This paradigm allows expressing the perceived intensity as a function of stimulus intensity. Overall, a linear function provided a good approximation of the data. In early research on the relation between stimuli and perception, it was argued that the psychometric function that relates perceived to physical intensity should follow a logarithmic law [43] or a power law [44]. However, for a narrow range of stimuli, such as in the present experiments, the curvature of the function can be negligible, and a straight line can provide a reasonable approximation of the data [41]. In the longitudinal case, the slope coefficient of the fitted function was increased by a factor 1.3 when the ASB system was active (i.e., from $[\beta_{i_{ME}}]=29.95$ to $[\beta_{i_{ME}}+\beta_{ci_{ME}}]=39.75$). In this condition, somatosensory stimulation was primarily provided by the belts. This finding suggests that the belts provide somatosensory cues that can effectively augment the inertial stimulation for simulation of sustained decelerations in dynamic driving simulators.

In addition to the ME task, participants performed a 2-Interval Forced Choice task (2IFC). In this task, participants discriminate stimuli on the basis of their intensity. Although the binary data obtained from 2IFC tasks do not provide information about the size of the perceived intensity nor on the shape of the distribution of underlying percepts (as in the ME task), the binary responses on 2IFC tasks are not affected by cognitive strategies such as rounding off to the nearest decade and are not affected by the scaling of the internal representation of acceleration intensity. Consequently, the slope of the psychometric function fitted to the data in this task may be considered a more objective measurement of perceptual precision. The model fits for this data indicated that the slope was steeper in conditions with active somatosensory stimulation. For the lateral and longitudinal maneuvers, the slopes were approximately a factor 2 and a factor 1.5 steeper when there was active somatosensory stimulation (lateral: $\left[\beta_{i_{2IFC}}+\beta_{mi_{2IFC}}\right]=1.17$ vs. $[\beta_{i_{2IFC}}+\beta_{mi_{2IFC}}+\beta_{ci_{2IFC}}]=2.35$; longitudinal: $[\beta_{i_{2IFC}}]=2.41$ vs. $[\beta_{i_{2IFC}}+\beta_{ci_{2IFC}}]=3.58$). These findings thus indicate that the precision of the perceived intensity was higher when somatosensory information was present than when it was not. The direction of this effect, namely a reduction of perceptual uncertainty when more senses are stimulated, is consistent with predictions of psychophysical models of multisensory perception that state that information provided by multiple sensory systems is integrated in a statistically optimal fashion [16, 17, 23, 27, 28, 30, 45, 46]. When the precision of each contributing sensory system is known, these models can make exact predictions on the weights attributed to information provided by the different sensory systems and on the perceptual precision for combined multisensory conditions. To make such predictions for the present paradigm would require additional ME and 2IFC data for ASB stimulation in isolation from the other senses; without visual or inertial stimulation. Although it would be theoretically possible to collect this data, such an experimental condition (or isolated inertial stimulation, for that matter) would not bear any resemblance to a driving simulation, and is therefore beyond the scope of the present study. A consequence of this is that the present data do not allow us to evaluate the exact predictions of these perception models. This also means that on the basis of the present statistical evaluations of the data, the possibility cannot be excluded that the additional somatosensory cues were processed independently. There is however evidence to argue against this possibility: first, such processing would not be consistent with the subjective experiences reported by our participants during debriefing: if the cues had been processed independently, this would produce an unnatural situation where a somatosensory sensation co-exists with an inertial sensation, whereas our participants all indicated that adding the somatosensory cues actually improved simulation realism; and second, a number of studies show that cues of a somatosensory nature are merged with visual and vestibular cues for other aspects of self-motion and orientation perception. For instance, podokinetic cues (stepping) are combined with vestibular cues to generate vection during active turning [47, 48]. Moreover, it has been shown that the somatosensory cues are combined with visual and vestibular cues for spatial orientation [49] and postural sway [50].

Finally, it is important to note two potential limitations of the present study. First, the findings were obtained in the context of a dynamic driving simulator, and the ASB were tuned specifically to add to the physical cues provided by the motion base. Consequently, the findings may not generalize to fixed-base simulators. These simulators do not provide the physical motion cues that can be generated in dynamic driving simulators and can thus be expected to result in different sensory weightings. Second, the data were obtained from a limited sample of participants. However, such small numbers of participants are actually not unusual in studies using psychophysical procedures to quantify psychological effects. This may be justified by the notion that the low-level (neural) mechanisms responsible for perception do not typically differ between people, thus allowing generalizability of the results to the population [51–55]. To deal with the limited sample size in the best way, we chose to pool the data from the braking (four participants) and cornering (six participants) maneuvers for the statistical analysis, and to use multilevel models. These models account for individual differences by including random effects. The fixed effects shown as solid lines in Figs 5 and 6 may thus be interpreted as an average performance, corrected for individual differences. We believe that this approach provides a reasonable characterization of general effects.

## 5 Conclusion

We conclude that the addition of somatosensory information to dynamic driving simulations increases the precision of intensity percepts and that it can augment inertial longitudinal acceleration cues. The direction of the findings on perceptual precision is consistent with predictions made by psychophysical theories on multisensory perception, which state that this information is integrated in a statistically optimal way. Combined with subjective reports obtained via questionnaire, the findings suggest that ASB can add to simulation fidelity in dynamic driving simulators.

## Supporting information

S1 Data Data file containing participants’ answers obtained during ME tasks. (MAT)

S2 Data Data file containing participants’ answers obtained during 2IFC tasks. (MAT)

S1 Questionnaire Data file containing participants’ answers obtained during debriefing phase. (XLSX)

S1 Appendix (PDF)
