# Supplementary material for: Assessing the contribution of active somatosensory stimulation to self-acceleration perception in dynamic driving simulators
Source: PLoS One. 2021 Nov 18;16(11):e0259015. doi: 10.1371/journal.pone.0259015 (PMC8601569; doi:10.1371/journal.pone.0259015)
Supplement: S1 Appendix — (PDF) [file pone.0259015.s004.pdf]

Appendix

Table 4. Main Motion Cueing parameters

Name	Value	Name	Value
Long. Acc. HP	0.7	Lat. Acc.	3
Long. Acc.	8.5	Roll Vel. HP	0
Pitch Vel. HP	0.1	Roll Vel. HP	0
Pitch Vel.	1	Roll Tilt LP	0.2
Pitch Tilt LP	0.2	Roll Tilt	0.3
Pitch Tilt	0.8	Yaw Vel. HP	0.35
Lat. Acc. HP	0.5	Yaw Vel.	0.8

Table 5. ASB parameters

Name	Value	Range	Unit
Global Gain	1.4	0-1.5	
Inferior Gain	0	0-1.5	
Inf. Gain NonLinear	1.1	0.7-1.5	
Inf. Preload	0	0-0.4	bar
Inf. Asimmetry Gain	0	0-0.5	
Lower Lateral Gain	0.3	0-1.5	
Low. Lat. Gain NL	1.1	0.7-1.5	
Low. Lat. Preload	0	0-0.4	bar
Upper Lat. Gain	1.5	0-1.5	
Up. Lat. Gain NL	1.1	0.7-1.5	
Up. Lat. Preload	0	0-0.4	bar
Rear gain	0.7	0-1.5	
Rear gain NL	1.1	0.7-1.5	
Rear Preload	0.11	0-0.4	bar
Rear Asimmetry Gain	0.1	0-0.5	
Longitudinal acc. LP	4	2-10	Hz
Lateral acc. LP	0.7	0.7-5	Hz
Vertical acc. LP	5	2-10	Hz
Pull Scale AB	23	10-25	Kg/g
Min Pull AB	1	0.5-1	Kg
Zero Pull (Preload) AB	1	1-2	Kg
Low pass frequency AB	2	2-5	Hz
